# A preoperative nomogram integrating transvaginal ultrasound endometrial thickness and nutritional, inflammatory, and metabolic indicators for predicting lymph node metastasis in endometrial cancer

**DOI:** 10.3389/fpubh.2026.1900875

**Published:** 2026-07-29

**Authors:** Xiaodi Xu, Ting Li, Gongxue Xian, Qiping Chen

**Affiliations:** 1 Department of Ultrasound, The Fourth Affiliated Hospital of Soochow University (Suzhou Dushu Lake Hospital), Suzhou, Jiangsu, China; 2 Department of Gynecology and Pediatrics, No. 965 Hospital of the PLA Joint Logistics Support Force, Jilin City, Jilin, China; 3 Department of Outpatient, The General Hospital of Western Theater Command of Chinese People's Liberation Army, Chengdu, Sichuan, China

**Keywords:** endometrial cancer, endometrial thickness, lymph node metastasis, neutrophil-to-lymphocyte ratio, nomogram

## Abstract

**Background:**

Preoperative assessment of lymph node metastasis (LNM) risk is critical for surgical planning in endometrial cancer (EC). Transvaginal ultrasound (TVU) endometrial thickness (ET) is a routinely available morphological parameter, whereas nutritional and metabolic indicators reflect the systemic host-tumor interaction. Although prior studies have incorporated either imaging or biomarker-based predictors individually, a unified preoperative framework integrating TVU morphology with a comprehensive panel of nutritional, inflammatory, and metabolic biomarkers for LNM prediction has not been established.

**Methods:**

This retrospective cohort study enrolled 203 patients with histopathologically confirmed EC who underwent definitive surgical staging at our institution between January 2021 and December 2025. Preoperative TVU-measured ET, tumor size, depth of myometrial invasion (MI), and cervical stromal invasion were recorded, alongside nutritional, inflammatory, and metabolic biomarkers including CA-125, HE4, albumin, neutrophil-to-lymphocyte ratio (NLR), platelet-to-lymphocyte ratio (PLR), prognostic nutritional index (PNI), fibrinogen, fasting blood glucose, and triglyceride-glucose (TyG) index. Spearman correlation analysis quantified associations between ET and biomarkers. Univariate and multivariate logistic regression identified independent LNM predictors. Receiver operating characteristic (ROC) curve analysis with DeLong tests evaluated discriminative performance, and a nomogram was constructed and internally validated.

**Results:**

LNM was confirmed in 35 patients (17.2%). ET was significantly higher in LNM-positive patients (34.7 ± 11.6 mm vs. 22.4 ± 8.1 mm, *p* < 0.001) and demonstrated moderate-to-strong positive correlations with CA-125 (*ρ* = 0.512), HE4 (*ρ* = 0.468), NLR (*ρ* = 0.398), fibrinogen (*ρ* = 0.426), and TyG index (*ρ* = 0.233), and negative correlations with albumin (*ρ* = −0.374) and PNI (*ρ* = −0.441) (all *p* < 0.001). Multivariate analysis identified ET (adjusted OR = 1.092 per mm, 95% CI: 1.043–1.143), deep MI ≥ 50% (OR = 4.827), CA-125 ≥ 35 U/mL (OR = 3.614), NLR ≥ 3.0 (OR = 3.082), PNI < 50 (OR = 2.947), fibrinogen ≥4.0 g/L (OR = 2.614), grade 3 histology (OR = 3.408), and non-endometrioid type (OR = 2.891) as independent predictors of LNM (all *p* < 0.05). The combined nomogram achieved an AUC of 0.941 (95% CI: 0.895–0.987), significantly outperforming ET alone (AUC = 0.812, *p* < 0.001).

**Conclusion:**

Preoperative TVU-ET is significantly correlated with nutritional, inflammatory, and metabolic indicators and independently predicts LNM in EC. A nomogram integrating ET with CA-125, NLR, PNI, fibrinogen, tumor grade, and histological type provides promising, good internally validated discrimination for LNM and may guide individualized surgical planning. External validation in independent cohorts remains necessary before clinical implementation.

## 1 Introduction

Endometrial cancer (EC) is the most prevalent gynecological malignancy in high-income countries and ranks among the leading causes of cancer-related mortality in women worldwide. According to GLOBOCAN 2020 estimates, approximately 417,367 new EC cases and 97,370 deaths were recorded globally, with incidence rates continuing to rise, largely driven by aging populations, obesity epidemics, and metabolic dysregulation (1). In China, EC has now surpassed cervical cancer as the most common gynecological cancer in urban settings, accounting for substantial disease burden and healthcare costs (2).

Lymph node metastasis (LNM) is a critical determinant of surgical staging, adjuvant therapy planning, and long-term prognosis in EC. Patients with LNM require systematic pelvic and para-aortic lymphadenectomy, sentinel lymph node biopsy, and often combination adjuvant chemotherapy with radiation, whereas those with pathologically node-negative disease confined to the uterus may be managed with less extensive surgery and limited adjuvant treatment. Accurate preoperative identification of patients at high risk for LNM is therefore fundamental to avoiding unnecessary lymph node dissection—with its attendant risks of lymphedema and prolonged operative time—while ensuring adequate oncological staging in high-risk cases.

Transvaginal ultrasound (TVU) is the first-line imaging modality recommended for preoperative evaluation of EC, offering the ability to assess endometrial thickness (ET), tumor dimensions, depth of myometrial invasion (MI), and cervical stromal involvement in a non-invasive, widely accessible, and cost-effective manner (3–5). Prior studies have demonstrated that TVU-measured ET and the extent of MI correlate with tumor grade and FIGO stage, but its independent capacity to predict LNM when combined with systemic biomarkers has been insufficiently explored (6, 7). A 2025 systematic review confirmed that TVU assessment of myometrial and cervical invasion provides clinically useful staging information, particularly in resource-limited settings where MRI availability is constrained (4).

Nutritional and metabolic status is increasingly recognized as modifiable factors that shape the tumor microenvironment, host immune competence, and ultimately treatment response in EC. Malnutrition impairs lymphocyte function and heightens systemic inflammation, fostering conditions permissive to tumor progression and lymphatic spread. Composite nutritional, inflammatory, and metabolic indices—including the prognostic nutritional index (PNI), neutrophil-to-lymphocyte ratio (NLR), platelet-to-lymphocyte ratio (PLR), and fibrinogen—have each been individually associated with FIGO stage, LVSI, and survival outcomes in EC (8, 9). Several studies have previously combined imaging parameters or serological markers with inflammatory-nutritional indices for LNM prediction in EC (10, 11). However, a unified preoperative framework that simultaneously integrates TVU morphological findings with a comprehensive panel of nutritional, inflammatory, and metabolic biomarkers and evaluates their joint predictive value for LNM through a validated nomogram remains to be established.

The metabolic-inflammatory-nutritional score (MINS) proposed by Lin et al. (12) demonstrated that integrating metabolic, inflammatory, and nutritional parameters significantly improved LNM prediction in EC. Similarly, Yang et al. (13) showed that metabolic syndrome risk scores contribute incremental information to LNM nomograms beyond classic pathological variables. These findings collectively motivate integrating TVU morphology with nutritional, inflammatory, and metabolic biomarkers into a unified preoperative prediction framework.

The present retrospective cohort study was designed to (1) characterize the correlations between preoperative TVU-measured ET and a comprehensive panel of nutritional, inflammatory, and metabolic indicators in patients with EC; (2) identify independent preoperative predictors of LNM through multivariate logistic regression; and (3) develop and internally validate a nomogram combining TVU morphological parameters with nutritional, inflammatory, and metabolic biomarkers for individualized LNM risk stratification. This work aligns with the growing research agenda on nutrition and women’s cancers, aiming to translate nutritional biomarker evidence into actionable perioperative tools.

## 2 Materials and methods

### 2.1 Study design and patient selection

This retrospective cohort study was conducted at The Fourth Affiliated Hospital of Soochow University (Suzhou Dushu Lake Hospital), a tertiary gynecological oncology center in China, and included patients who underwent primary surgical staging for histopathologically confirmed EC between January 2021 and December 2025. The study was approved by the Institutional Ethics Committee (This is a single-center study; co-authors affiliated with other institutions contributed to data analysis and manuscript preparation, but all patient data were collected exclusively at this institution. All patients meeting the eligibility criteria during the study period (January 2021 to December 2025) were included consecutively without further selection. The Fourth Affiliated Hospital of Soochow University (Suzhou Dushu Lake Hospital) Approval No: 2026 Lunyan Pi No. 260178) and conducted in accordance with the Declaration of Helsinki. The requirement for individual informed consent was waived given the retrospective nature of the study, in accordance with local regulatory guidance.

Inclusion criteria were: (1) primary EC confirmed by endometrial biopsy or curettage prior to surgery; (2) complete preoperative TVU examination with recorded ET, tumor dimensions, and qualitative assessment of MI depth and cervical involvement; (3) complete preoperative laboratory data including CA-125, HE4, full blood count with differential, serum albumin, fibrinogen, fasting blood glucose, and fasting lipid profile; (4) definitive surgical staging including total hysterectomy with bilateral salpingo-oophorectomy and systematic pelvic ± para-aortic lymphadenectomy or sentinel lymph node biopsy; and (5) complete postoperative pathological data. For the purpose of the nomogram, tumor grade and histological subtype were ascertained from the preoperative endometrial biopsy or curettage specimen in all patients and were therefore available before surgery. We acknowledge that concordance between preoperative biopsy-derived grade and final surgical pathology is imperfect, with upgrade rates of approximately 15–20% reported in the literature; this limitation is discussed further in the limitations section.

Exclusion criteria were: (1) synchronous or prior malignancy; (2) preoperative chemotherapy or radiotherapy; (3) pregnancy; (4) active infection or systemic inflammatory disease within 30 days of surgery (conditions known to confound inflammatory indices); (5) incomplete TVU or laboratory data; and (6) patients who received neoadjuvant therapy or for whom lymph node assessment was not performed.

**Figure 1:**
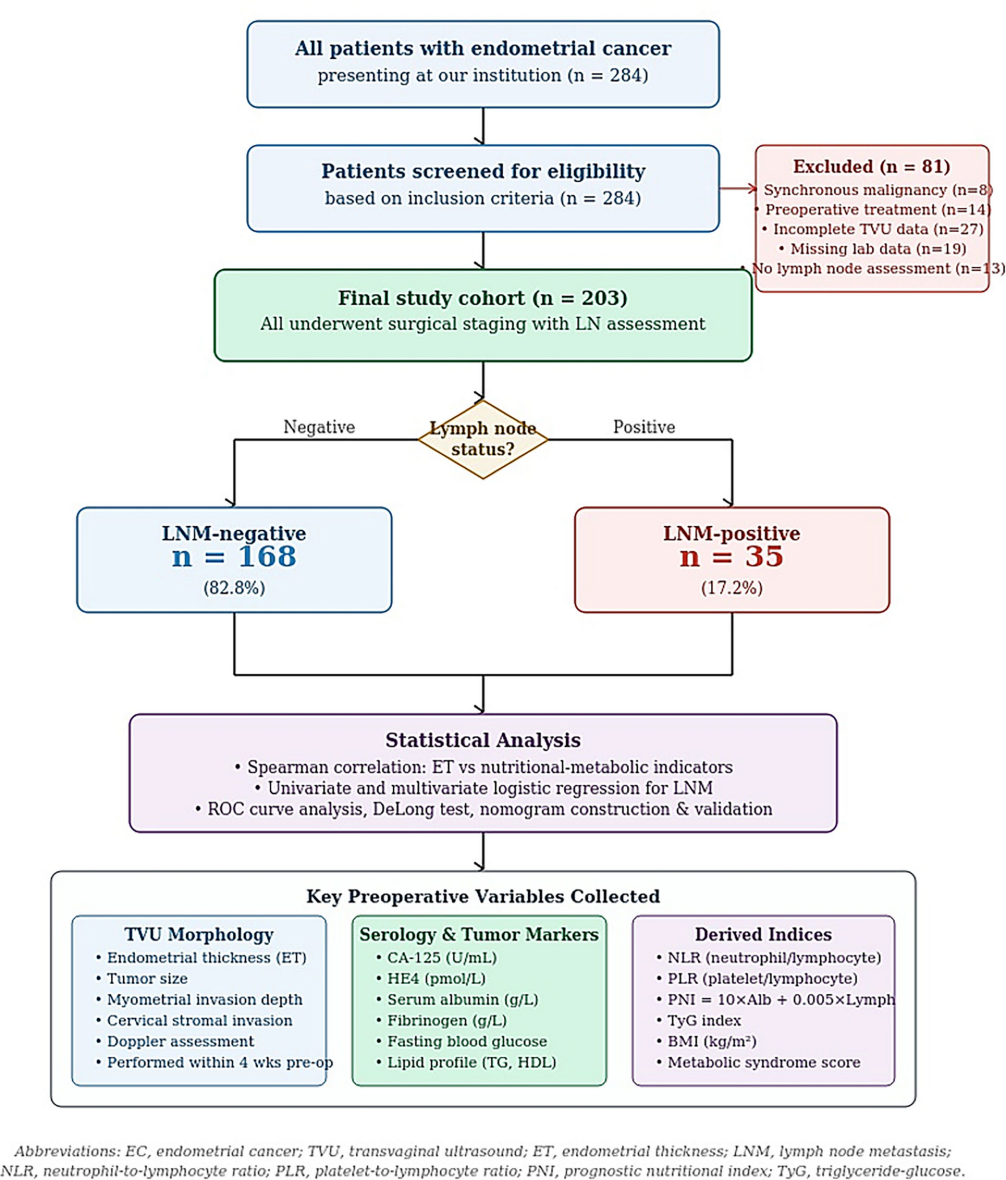
Flowchart of patient selection and study design. EC, endometrial cancer; TVU, transvaginal ultrasound; ET, endometrial thickness; LNM, lymph node metastasis; NLR, neutrophil-to-lymphocyte ratio; PNI, prognostic nutritional index.

After applying these criteria, 203 patients were eligible for analysis (Figure 1—Study Flowchart).

### 2.2 Transvaginal ultrasound assessment

All TVU examinations were performed by experienced gynecological sonographers using standardized equipment (GE Voluson E10, GE Healthcare, Chicago, IL, United States) within 4 weeks before surgery. ET was measured at the thickest point in the sagittal plane, perpendicular to the endometrial midline echo, as the full double-layer endometrial thickness in millimeters. Tumor size was defined as the largest dimension of the endometrial mass measured on TVU. Depth of myometrial invasion was classified as superficial (<50%) or deep (≥50%) based on real-time gray-scale and Doppler assessment. Cervical stromal invasion was documented as present or absent. All TVU findings were prospectively recorded in the electronic medical record at the time of the examination and retrospectively extracted for this study.

### 2.3 Laboratory parameters and nutritional-metabolic indicators

All blood samples were obtained within 1 week before surgery under standardized fasting conditions. The following parameters were collected from the electronic laboratory system: serum CA-125 (U/mL), HE4 (pmol/L), serum albumin (g/L), total white blood cell count with differential (neutrophil, lymphocyte, platelet counts), fibrinogen (g/L), fasting blood glucose (mmol/L), triglycerides (mmol/L), and high-density lipoprotein cholesterol (mmol/L).

Derived nutritional and metabolic indices were calculated as follows: NLR = absolute neutrophil count / absolute lymphocyte count; PLR = platelet count / absolute lymphocyte count; PNI = 10 × serum albumin (g/dL) + 0.005 × total lymphocyte count (per mm^3^), as originally described; TyG index = ln [triglycerides (mg/dL) × fasting plasma glucose (mg/dL) / 2]. BMI was calculated as weight (kg) divided by height squared (m^2^) and classified using Asian-specific cutoffs (overweight/obesity ≥23 kg/m^2^).

### 2.4 Pathological assessment and outcome definition

All surgical specimens were evaluated by dedicated gynecological pathologists blinded to preoperative imaging findings. Histological type (endometrioid vs. non-endometrioid), tumor grade (G1–G3 per 2014 WHO classification), depth of myometrial invasion, cervical stromal invasion, lymphovascular space invasion (LVSI), and peritoneal cytology were recorded. Lymph node status was determined by systematic pathological examination of all dissected nodes. LNM was defined as histopathologically confirmed tumor deposit in any pelvic or para-aortic lymph node. Surgical staging was assigned according to the 2023 FIGO staging system for endometrial carcinoma (14).

### 2.5 Statistical analysis

Continuous variables were summarized as mean ± standard deviation (SD) or median (interquartile range [IQR]) as appropriate after testing for normality with the Shapiro–Wilk test. Categorical variables were expressed as frequencies and percentages. Between-group comparisons (LNM-positive vs. LNM-negative) were performed using the independent samples *t*-test or Mann–Whitney *U* test for continuous variables, and the chi-square or Fisher’s exact test for categorical variables. Specifically, CA-125, HE4, fibrinogen, and related laboratory markers demonstrating non-normal distributions are reported as median (IQR) and compared using the Mann–Whitney U test; normally distributed variables are reported as mean ± SD and compared using the independent samples t-test.

Spearman rank correlation coefficients (*ρ*) with 95% confidence intervals were computed to quantify associations between preoperative ET and each nutritional, inflammatory, and metabolic indicator. Univariate logistic regression was applied to all candidate predictors, and variables with *p* < 0.10 were entered into multivariate logistic regression with backward elimination. Variance inflation factors (VIF < 5) confirmed absence of clinically meaningful multicollinearity. Binary cutoffs for continuous predictors were applied as follows: the CA-125 threshold of 35 U/mL was adopted from established clinical guidelines and prior literature; the ET cutoff of 28.5 mm was identified by Youden index in the present cohort and is consistent with values reported in previous studies; the thresholds for NLR (≥3.0), PNI (<50), and fibrinogen (≥4.0 g/L) were derived from ROC analysis within the current dataset and should therefore be interpreted with appropriate caution. To assess whether the findings were threshold-dependent, a sensitivity analysis modelling these variables as continuous predictors was also performed. As a further sensitivity analysis addressing potential overfitting given the events-per-variable ratio of approximately 4.4, LASSO-penalised logistic regression was applied as an alternative variable selection approach. ROC curves were constructed for each individual predictor and for combined models, and AUC differences were compared using the DeLong method. A nomogram integrating the independent predictors identified in multivariate analysis was constructed using the rms package in R (version 4.3.2). Internal validation was performed using 1,000-iteration bootstrap resampling; the apparent AUC, optimism estimate, and optimism-corrected C-index are reported separately. Calibration was assessed with the Hosmer-Lemeshow goodness-of-fit test and a calibration plot displaying calibration slope and intercept. Subgroup analysis stratified patients by ET tertile to examine the gradient relationship between ET and LNM risk. All statistical tests were two-tailed, and *p* < 0.05 was considered statistically significant. Analyses were performed in R 4.3.2 (R Foundation for Statistical Computing) and SPSS version 26.0 (IBM Corp., Armonk, NY). A complete-case analysis was employed; patients with missing data in any preoperative TVU parameter or laboratory variable specified in the inclusion criteria were excluded during patient selection, as detailed in Section 2.1. No imputation was performed.

## 3 Results

### 3.1 Baseline characteristics

A total of 203 patients met the eligibility criteria and were included in the final analysis. The mean age was 56.4 ± 9.2 years (range 32–78 years) and the mean BMI was 26.8 ± 4.3 kg/m^2^. The majority of patients were postmenopausal (*n* = 151, 74.4%). Endometrioid histology predominated (*n* = 176, 86.7%). LNM was confirmed in 35 patients (17.2%). Patients with LNM were significantly more likely to have higher grade (G3: 54.3% vs. 13.7%, *p* < 0.001), non-endometrioid histology (31.4% vs. 9.5%, *p* = 0.001), and higher BMI (29.2 ± 4.7 vs. 26.3 ± 4.1 kg/m^2^, *p* = 0.001) compared with LNM-negative patients. Baseline characteristics are summarized in Table 1.

**Table 1:** Baseline characteristics of patients stratified by lymph node metastasis status.

Variable	Total (*n* = 203)	LNM− (*n* = 168)	LNM+ (*n* = 35)	*p* value
Age (years)	56.4 ± 9.2	55.9 ± 9.0	59.1 ± 9.8	0.071
BMI (kg/m^2^)	26.8 ± 4.3	26.3 ± 4.1	29.2 ± 4.7	0.001
Postmenopausal, *n* (%)	151 (74.4)	122 (72.6)	29 (82.9)	0.198
Diabetes mellitus, *n* (%)	68 (33.5)	52 (31.0)	16 (45.7)	0.093
Hypertension, *n* (%)	84 (41.4)	67 (39.9)	17 (48.6)	0.352
Histological type				
Endometrioid	176 (86.7)	152 (90.5)	24 (68.6)	0.001
Non-endometrioid	27 (13.3)	16 (9.5)	11 (31.4)	
Tumor grade				
G1	89 (43.8)	82 (48.8)	7 (20.0)	<0.001
G2	72 (35.5)	63 (37.5)	9 (25.7)	
G3	42 (20.7)	23 (13.7)	19 (54.3)	
FIGO stage				
Stage I	138 (68.0)	125 (74.4)	13 (37.1)	<0.001
Stage II	27 (13.3)	24 (14.3)	3 (8.6)	
Stage III	38 (18.7)	19 (11.3)	19 (54.3)	

LNM, lymph node metastasis; BMI, body mass index; FIGO, international federation of gynecology and obstetrics. Data are presented as mean ± SD or n (%). *p* values from independent *t*-test or chi-square test as appropriate.

### 3.2 Comparison of preoperative TVU and nutritional-metabolic parameters

Compared with LNM-negative patients, those with confirmed LNM exhibited significantly greater mean ET (34.7 ± 11.6 mm vs. 22.4 ± 8.1 mm, *p* < 0.001), larger tumor size on TVU (5.1 ± 1.8 cm vs. 3.2 ± 1.4 cm, *p* < 0.001), higher rates of deep MI ≥ 50% (74.3% vs. 27.4%, *p* < 0.001), and more frequent cervical stromal invasion (40.0% vs. 10.7%, *p* < 0.001). LNM-positive patients also demonstrated markedly elevated CA-125 (87.6 ± 62.4 U/mL vs. 24.3 ± 18.7 U/mL) and HE4 (184.3 ± 97.5 pmol/L vs. 96.4 ± 38.2 pmol/L), higher NLR (3.47 ± 1.26 vs. 2.14 ± 0.82), PLR (189.4 ± 67.8 vs. 128.6 ± 42.3), and fibrinogen (4.38 ± 1.14 g/L vs. 3.12 ± 0.78 g/L), along with lower albumin (37.6 ± 4.9 g/L vs. 42.1 ± 3.8 g/L) and PNI (45.8 ± 7.4 vs. 54.2 ± 6.1) (all *p* < 0.001). Detailed comparisons are presented in Table 2.

**Table 2:** Comparison of preoperative TVU findings and nutritional-metabolic parameters between LNM-negative and LNM-positive patients.

Parameter	LNM− (*n* = 168)	LNM+ (*n* = 35)	*p* value
ET by TVU (mm)	22.4 ± 8.1	34.7 ± 11.6	<0.001
Tumor size by TVU (cm)	3.2 ± 1.4	5.1 ± 1.8	<0.001
Deep MI (≥50%) on TVU, *n* (%)	46 (27.4)	26 (74.3)	<0.001
Cervical stromal invasion on TVU, *n* (%)	18 (10.7)	14 (40.0)	<0.001
CA-125 (U/mL)	24.3 ± 18.7	87.6 ± 62.4	<0.001
HE4 (pmol/L)	96.4 ± 38.2	184.3 ± 97.5	<0.001
Albumin (g/L)	42.1 ± 3.8	37.6 ± 4.9	<0.001
NLR	2.14 ± 0.82	3.47 ± 1.26	<0.001
PLR	128.6 ± 42.3	189.4 ± 67.8	<0.001
PNI	54.2 ± 6.1	45.8 ± 7.4	<0.001
Fibrinogen (g/L)	3.12 ± 0.78	4.38 ± 1.14	<0.001
Fasting blood glucose (mmol/L)	5.64 ± 1.12	7.21 ± 1.98	<0.001
TyG index	8.61 ± 0.74	9.14 ± 0.93	<0.001

ET, endometrial thickness; TVU, transvaginal ultrasound; MI, myometrial invasion; CA-125, carbohydrate antigen 125; HE4, human epididymis protein 4; NLR, neutrophil-to-lymphocyte ratio; PLR, platelet-to-lymphocyte ratio; PNI, prognostic nutritional index; TyG, triglyceride-glucose. Data are mean ± SD or n (%).

### 3.3 Correlation between endometrial thickness and nutritional-metabolic indicators

**Figure 2:**
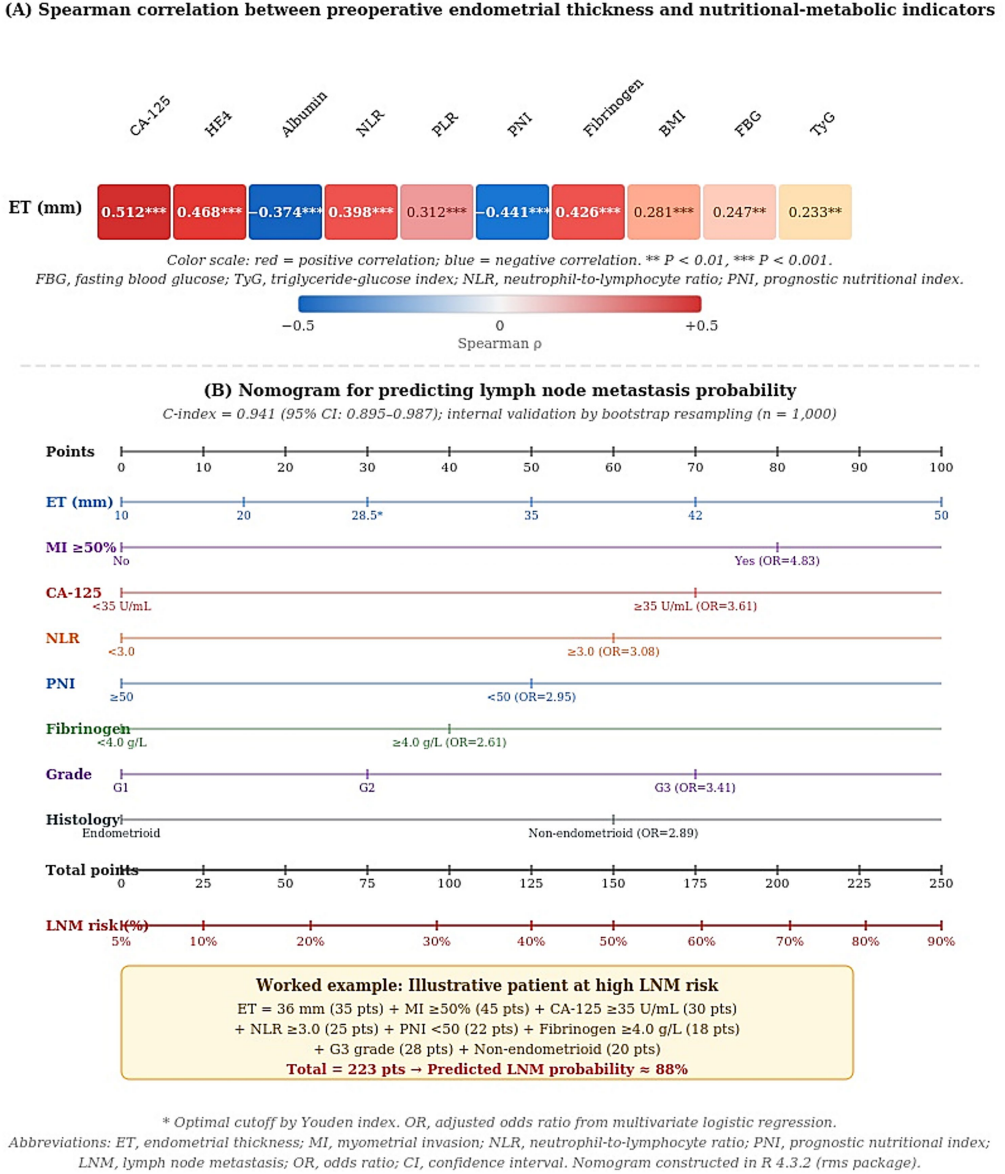
Correlation analysis and nomogram for predicting lymph node metastasis. **(A)** Spearman correlation heatmap between preoperative endometrial thickness and nutritional, inflammatory, and metabolic indicators, including CA-125, HE4, albumin, NLR, PLR, PNI, fibrinogen, BMI, fasting blood glucose, and TyG index. Color scale indicates correlation coefficient (*ρ*): red = positive, blue = negative. **(B)** Nomogram integrating endometrial thickness, deep myometrial invasion, CA-125, NLR, PNI, fibrinogen, tumor grade, and histological type for individualized prediction of lymph node metastasis probability. Each predictor is assigned a score on the top scale; scores are summed to yield total points, which map to the probability of LNM on the bottom axis. Calibration of the nomogram was confirmed using bootstrap resampling (1,000 iterations); the optimism-corrected C-index was 0.941 (95% CI: 0.895–0.987). BMI, body mass index; TyG, triglyceride-glucose; NLR, neutrophil-to-lymphocyte ratio; PNI, prognostic nutritional index; LNM, lymph node metastasis.

Spearman correlation analysis demonstrated that preoperative ET was significantly and positively correlated with CA-125 (*ρ* = 0.512, *p* < 0.001), HE4 (*ρ* = 0.468, *p* < 0.001), NLR (*ρ* = 0.398, *p* < 0.001), fibrinogen (*ρ* = 0.426, *p* < 0.001), PLR (*ρ* = 0.312, *p* < 0.001), BMI (*ρ* = 0.281, *p* < 0.001), fasting blood glucose (*ρ* = 0.247, *p* = 0.001), and TyG index (*ρ* = 0.233, *p* = 0.002). Conversely, ET was significantly and negatively correlated with albumin (*ρ* = −0.374, *p* < 0.001) and PNI (*ρ* = −0.441, *p* < 0.001). These findings indicate that increasing ET reflects not only tumor bulk but also systemic nutritional depletion, inflammatory activation, and metabolic dysregulation. It should be noted that these Spearman analyses are descriptive and may partly reflect shared associations with tumor burden, grade, and depth of myometrial invasion; the multivariate logistic regression model, which adjusts for these covariates simultaneously, provides a more appropriate basis for assessing independent predictor contributions. Results are presented in Table 3 and illustrated in Figure 2A.

**Table 3:** Spearman correlation between preoperative endometrial thickness and nutritional-metabolic indicators.

Indicator	r/*ρ*	95% CI	*P* value
CA-125 (U/mL)	0.512	0.401–0.611	<0.001
HE4 (pmol/L)	0.468	0.353–0.572	<0.001
Albumin (g/L)	−0.374	−0.484−−0.254	<0.001
NLR	0.398	0.280–0.506	<0.001
PLR	0.312	0.186–0.428	<0.001
PNI	−0.441	−0.547−−0.323	<0.001
Fibrinogen (g/L)	0.426	0.310–0.531	<0.001
BMI (kg/m^2^)	0.281	0.153–0.400	<0.001
Fasting blood glucose (mmol/L)	0.247	0.116–0.368	0.001
TyG index	0.233	0.101–0.356	0.002

NLR, neutrophil-to-lymphocyte ratio; PLR, platelet-to-lymphocyte ratio; PNI, prognostic nutritional index; TyG, triglyceride-glucose index. *ρ*, Spearman rank correlation coefficient. 95% CI, 95% confidence interval.

### 3.4 Univariate and multivariate logistic regression for lymph node metastasis

On univariate logistic regression, all TVU parameters (ET, tumor size, deep MI, cervical invasion), serological markers (CA-125, HE4, albumin, fibrinogen), and inflammatory-nutritional indices (NLR, PLR, PNI) were significantly associated with LNM (all *p* < 0.001). BMI, tumor grade G3, and non-endometrioid histology were also significant univariate predictors (Table 4).

**Table 4:** Univariate logistic regression analysis for lymph node metastasis.

Variable	OR	95% CI	*P* value
ET by TVU (per mm)	1.124	1.081–1.169	<0.001
Tumor size by TVU (per cm)	1.872	1.491–2.351	<0.001
Deep MI ≥ 50% (yes vs. no)	7.854	3.508–17.574	<0.001
Cervical stromal invasion (yes)	5.600	2.416–12.985	<0.001
CA-125 (per U/mL)	1.038	1.026–1.051	<0.001
HE4 (per pmol/L)	1.009	1.006–1.013	<0.001
Albumin (per g/L)	0.878	0.823–0.937	<0.001
NLR (per unit)	2.741	1.962–3.830	<0.001
PLR (per unit)	1.013	1.007–1.019	<0.001
PNI (per unit)	0.852	0.806–0.901	<0.001
Fibrinogen (per g/L)	3.167	1.994–5.027	<0.001
BMI (per kg/m^2^)	1.163	1.059–1.277	0.001
Tumor grade G3 (vs. G1)	4.982	1.912–12.974	0.001
Non-endometrioid (vs. endometrioid)	4.367	1.876–10.165	<0.001

OR, odds ratio; CI, confidence interval; ET, endometrial thickness; MI, myometrial invasion; TVU, transvaginal ultrasound; NLR, neutrophil-to-lymphocyte ratio; PLR, platelet-to-lymphocyte ratio; PNI, prognostic nutritional index.

Following multivariate logistic regression with backward stepwise elimination, eight variables independently predicted LNM: ET (adjusted OR = 1.092, 95% CI: 1.043–1.143, per mm), deep MI ≥ 50% (OR = 4.827, *p* = 0.002), CA-125 ≥ 35 U/mL (OR = 3.614, *p* = 0.009), NLR ≥ 3.0 (OR = 3.082, *p* = 0.018), PNI < 50 (OR = 2.947, *p* = 0.028), fibrinogen ≥4.0 g/L (OR = 2.614, *p* = 0.047), tumor grade G3 (OR = 3.408, *p* = 0.023), and non-endometrioid histology (OR = 2.891, *p* = 0.049). Multivariate results are summarized in Table 5. The resulting nomogram is shown in Figure 2B.

**Table 5:** Multivariate logistic regression analysis for lymph node metastasis.

Variable	Adjusted OR	95% CI	*P* value
ET by TVU (per mm)	1.092	1.043–1.143	<0.001
Deep MI ≥ 50% (yes)	4.827	1.814–12.846	0.002
CA-125 ≥ 35 U/mL (yes)	3.614	1.371–9.532	0.009
NLR ≥ 3.0 (yes)	3.082	1.218–7.799	0.018
PNI < 50 (yes)	2.947	1.124–7.727	0.028
Fibrinogen ≥ 4.0 g/L (yes)	2.614	1.012–6.753	0.047
Tumor grade G3 (yes)	3.408	1.185–9.797	0.023
Non-endometrioid (yes)	2.891	1.003–8.332	0.049

OR, odds ratio; CI, confidence interval; MI, myometrial invasion; NLR, neutrophil-to-lymphocyte ratio; PNI, prognostic nutritional index. Backward stepwise elimination; VIF <5 for all retained variables.

### 3.5 ROC curve analysis and discriminative performance

**Figure 3:**
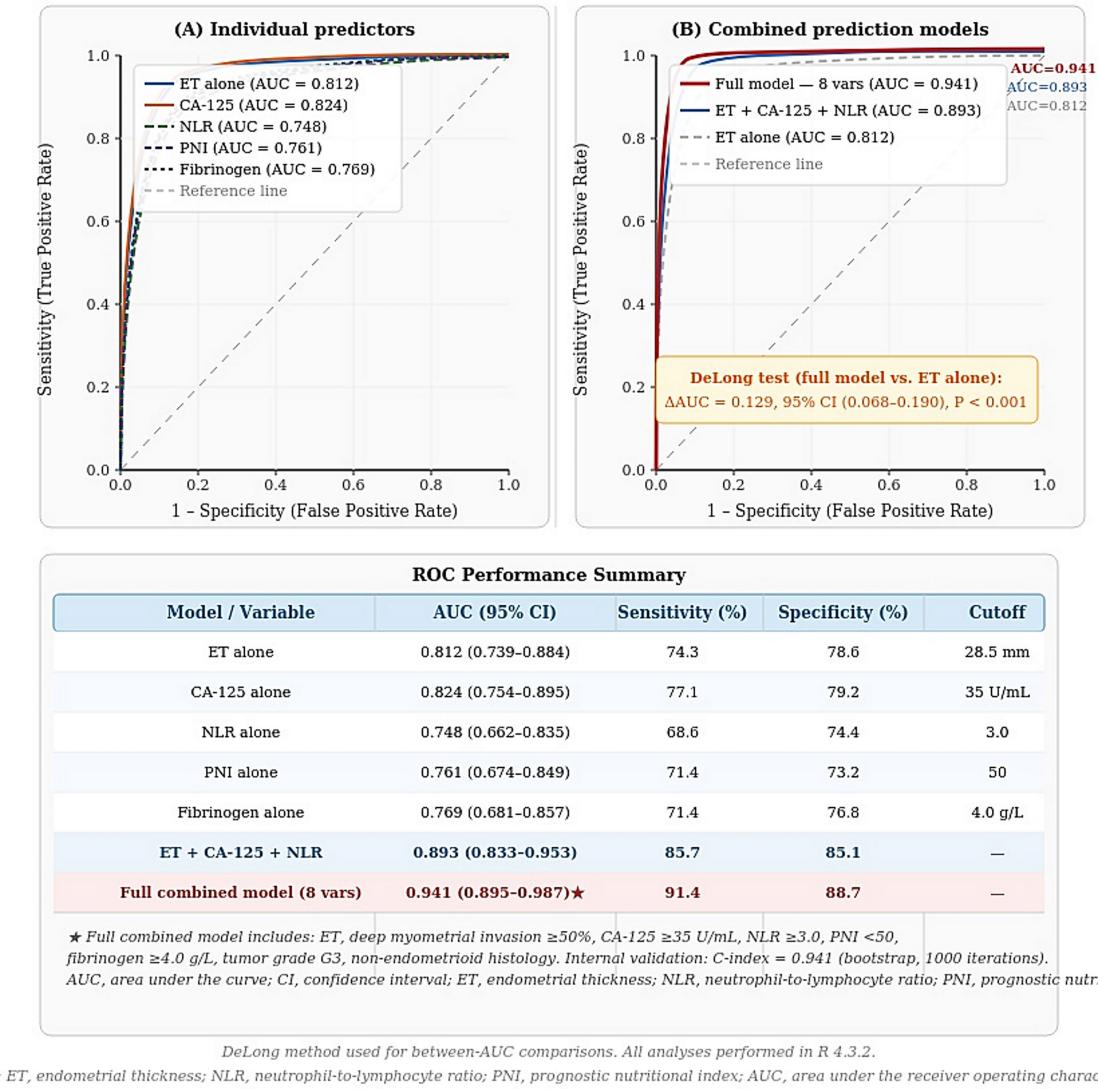
Receiver operating characteristic (ROC) curves for predicting lymph node metastasis. **(A)** Individual predictors: endometrial thickness (AUC = 0.812), CA-125 (AUC = 0.824), NLR (AUC = 0.748), PNI (AUC = 0.761), and fibrinogen (AUC = 0.769). **(B)** Combined models: ET + CA-125 + NLR (AUC = 0.893) versus the full combined nomogram incorporating all eight multivariate-identified predictors (ET, deep myometrial invasion, CA-125, NLR, PNI, fibrinogen, tumor grade, and histological type) (AUC = 0.941). DeLong test for AUC comparison between individual ET and combined model: *p* < 0.001. AUC, area under the curve; CI, confidence interval.

**Figure 4:**
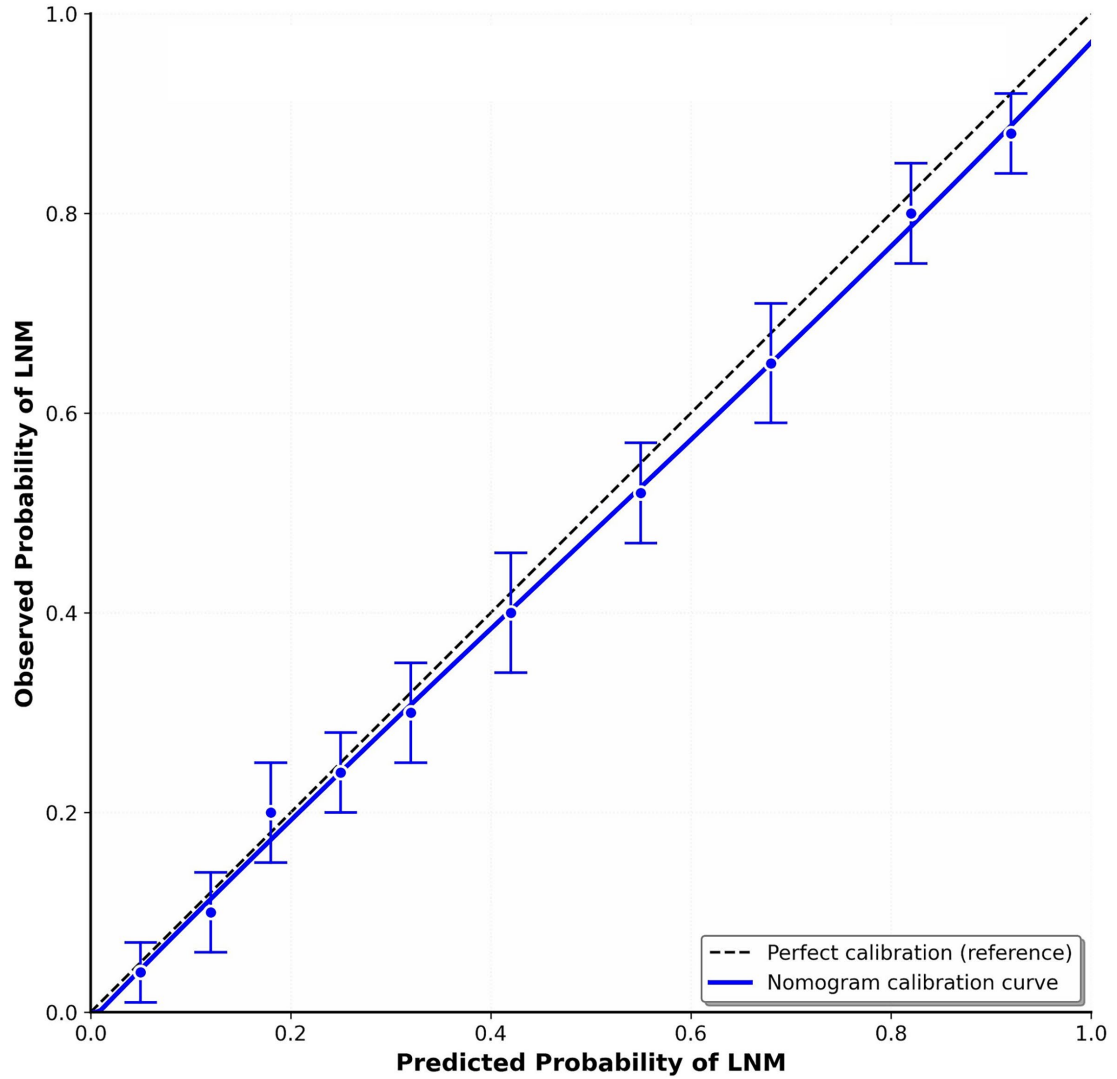
Calibration plot of the nomogram for predicting lymph node metastasis. The blue line and points with error bars represent the nomogram calibration results, and the black dashed line represents perfect calibration. LNM, lymph node metastasis.

ROC curve analysis demonstrated that ET alone achieved an AUC of 0.812 (95% CI: 0.739–0.884), with an optimal cutoff of 28.5 mm (sensitivity 74.3%, specificity 78.6%). CA-125 alone (AUC = 0.824) and PNI alone (AUC = 0.761) showed comparable individual performance. Combining ET with CA-125 and NLR improved the AUC to 0.893 (95% CI: 0.833–0.953), while the full combined nomogram incorporating all eight multivariate-identified predictors achieved an AUC of 0.941 (95% CI: 0.895–0.987), with sensitivity 91.4% and specificity 88.7%. DeLong test confirmed the combined model significantly outperformed ET alone (*p* < 0.001) and the three-variable model (*p* = 0.012). ROC performance metrics are summarized in Table 6 and illustrated in Figure 3. The calibration plot showed good agreement between the predicted and observed probabilities (Figure 4).

**Table 6:** ROC curve analysis for predicting lymph node metastasis: individual predictors and combined models.

Model/variable	AUC	95% CI	Sensitivity (%)	Specificity (%)	Cutoff
ET alone	0.812	0.739–0.884	74.3	78.6	28.5 mm
CA-125 alone	0.824	0.754–0.895	77.1	79.2	35 U/mL
NLR alone	0.748	0.662–0.835	68.6	74.4	3.0
PNI alone	0.761	0.674–0.849	71.4	73.2	50
Fibrinogen alone	0.769	0.681–0.857	71.4	76.8	4.0 g/L
ET + CA-125 + NLR	0.893	0.833–0.953	85.7	85.1	—
Combined model (all 8 predictors)	0.941	0.895–0.987	91.4	88.7	—

AUC, area under the curve; CI, confidence interval; ET, endometrial thickness; NLR, neutrophil-to-lymphocyte ratio; PNI, prognostic nutritional index. DeLong test used for AUC comparison between combined model and ET alone: *P* < 0.001.

### 3.6 Subgroup analysis by endometrial thickness tertile

Patients were stratified into three tertiles by ET: T1 (ET < 20 mm, *n* = 71), T2 (ET 20–30 mm, *n* = 81), and T3 (ET > 30 mm, *n* = 51). A clear gradient in LNM prevalence was observed across tertiles: 2.8% (T1), 9.9% (T2), and 49.0% (T3) (*p* < 0.001). Nutritional-metabolic indicators also worsened progressively across ET tertiles, with CA-125, NLR, and fibrinogen increasing and PNI decreasing from T1 to T3 (all *p* < 0.001). The rates of deep MI, grade G3, and non-endometrioid histology increased substantially with higher ET (Table 7). These findings demonstrate a consistent dose–response relationship between ET and LNM risk.

**Table 7:** Subgroup analysis by preoperative endometrial thickness tertile.

Variable	T1: ET <20 mm (*n* = 71)	T2: ET 20–30 mm (*n* = 81)	T3: ET >30 mm (*n* = 51)	*P* value
LNM, *n* (%)	2 (2.8)	8 (9.9)	25 (49.0)	<0.001
Deep MI (≥50%), *n* (%)	6 (8.5)	22 (27.2)	44 (86.3)	<0.001
CA-125 (U/mL)	18.4 ± 9.2	26.8 ± 14.7	64.3 ± 47.2	<0.001
NLR	1.92 ± 0.61	2.28 ± 0.78	3.51 ± 1.31	<0.001
PNI	56.3 ± 5.4	53.7 ± 6.2	46.2 ± 7.8	<0.001
Fibrinogen (g/L)	2.88 ± 0.61	3.24 ± 0.72	4.41 ± 1.02	<0.001
G3 tumor, n (%)	4 (5.6)	11 (13.6)	27 (52.9)	<0.001
Non-endometrioid, *n* (%)	3 (4.2)	7 (8.6)	17 (33.3)	<0.001

LNM, lymph node metastasis; MI, myometrial invasion; NLR, neutrophil-to-lymphocyte ratio; PNI, prognostic nutritional index. T1, ET <20 mm; T2, ET 20–30 mm; T3, ET >30 mm. *P* values from chi-square test or one-way ANOVA.

## 4 Discussion

This retrospective study of 203 patients with EC demonstrated four principal findings. First, preoperative TVU-measured ET was significantly elevated in LNM-positive patients and positively correlated with a broad spectrum of nutritional, inflammatory, and metabolic biomarkers, including CA-125, HE4, NLR, fibrinogen, and TyG index, while inversely correlating with albumin and PNI. Second, ET emerged as an independent predictor of LNM in multivariate analysis (adjusted OR = 1.092 per mm), alongside deep MI, CA-125, NLR, PNI, fibrinogen, tumor grade, and histological type. Third, the combined nomogram achieved promising, good internally validated discriminative performance (AUC = 0.941), substantially superior to any single predictor. Fourth, a clear dose–response relationship between ET tertile and LNM risk was confirmed, with rates rising from 2.8% in the lowest tertile to 49.0% in the highest Figure 4.

The strong positive correlation between ET and CA-125 (*ρ* = 0.512) is consistent with prior literature showing that increasing tumor bulk drives tumor antigen shedding and reflects greater local tumor burden. LyBarger et al. (15) confirmed CA-125 as an independent predictor of LNM in EC, and our data reinforce this finding with the added dimension that CA-125 elevation co-occurs with increasing ET as part of a broader biologically coherent phenotype of advanced local disease. The correlation between ET and HE4 (*ρ* = 0.468) is mechanistically plausible as HE4 expression in EC correlates with deep myometrial invasion and adverse molecular subtypes.

The inverse correlation between ET and PNI (*ρ* = −0.441) and albumin (*ρ* = −0.374) suggests that as tumors grow—reflected by thicker endometrium—the systemic nutritional status of the patient declines. The PNI, originally developed as a surrogate for preoperative immune-nutritional status, captures both hypoalbuminemia and lymphopenia, both of which reflect cancer-associated malnutrition and impaired anti-tumor immunity. A 2024 meta-analysis pooling 3,164 EC patients confirmed that low PNI is significantly associated with deep myometrial invasion, LVSI, advanced FIGO stage, and inferior survival (16–18). Our multivariate result (PNI < 50 independently predicting LNM, OR = 2.947) extends these associations to the domain of lymph node involvement and underscores the relevance of nutritional assessment in the preoperative EC work-up.

Fibrinogen—an acute-phase reactant and coagulation factor—emerged as an independent predictor of LNM (OR = 2.614 at ≥4.0 g/L cutoff). Elevated fibrinogen promotes tumor invasion and lymphatic metastasis by enhancing cell adhesion, facilitating tumor emboli formation within lymphatics, and sustaining a pro-tumorigenic fibrin matrix. Li et al. (19) developed a nomogram incorporating fibrinogen, albumin, NLR, and CA-125 for EC prognosis, providing strong support for fibrinogen’s relevance in this cancer, and Ronsini et al. (20) prospectively confirmed that NLR correlates with myometrial infiltration depth in stage I EC. The convergence of elevated ET with elevated fibrinogen and NLR suggests that high-risk EC patients exhibit a co-regulated phenotype of greater tumor burden, systemic inflammation, and impaired nutrition. Consistent with this, emerging evidence demonstrates that CD4 + T cell dysfunction in metabolic disease contexts—characterised by altered gene expression profiles—may further impair anti-tumour immunity, providing a mechanistic link between metabolic dysregulation, immune suppression, and tumour progression in EC (21).

The TyG index—a surrogate for insulin resistance reflecting the product of triglycerides and glucose—correlated modestly but significantly with ET (*ρ* = 0.233). Shi et al. (22) demonstrated that TyG index is associated with EC risk in a retrospective cohort, and Wang et al. (23) showed that metabolic disorders including hyperglycemia and obesity are associated with adverse clinicopathological features in EC. Insulin resistance facilitates tumor progression through activation of the insulin/IGF-1 and mTOR signaling axes, which are known drivers of endometrial carcinogenesis and invasion.

The MINS framework proposed by Lin et al. (12) similarly combined metabolic (TyG, cholesterol), inflammatory (NLR, SII), and nutritional (albumin) dimensions into a composite score predicting LNM and survival in EC, achieving AUC values of 0.79–0.83. Our combined model (AUC = 0.941) achieved superior discriminative performance, which may reflect the added contribution of preoperative TVU morphology—a dimension not included in the MINS. TVU provides direct tumor morphological information including ET and MI depth, which are biologically upstream determinants of LNM risk, whereas the MINS captures systemic host-tumor interaction. The integration of both layers—morphological and systemic—appears synergistic.

The proposed model does not currently incorporate molecular classification, including POLE mutational status and mismatch repair (MMR) proficiency, which are central to the 2021 ESGO/ESTRO/ESP risk stratification framework (24) and are increasingly available at the time of preoperative biopsy. This is a recognised limitation. Molecular subtype substantially influences lymph node metastasis risk—POLE-ultramutated tumours carry a favourable prognosis even with adverse morphological features, whereas p53-abnormal tumours are associated with a high risk of advanced disease (25). Future prospective studies should evaluate whether incorporating molecular classification alongside TVU and nutritional, inflammatory, and metabolic parameters further improves nomogram performance. In the meantime, the present nomogram is best understood as a complementary tool within a broader preoperative assessment framework that also includes molecular subtyping where available. Regarding nodal management, it should be noted that sentinel lymph node (SLN) mapping is increasingly adopted as the standard approach to nodal staging in endometrial cancer, offering comparable staging accuracy with reduced morbidity compared with systematic lymphadenectomy. The clinical utility of the nomogram should therefore be framed as supporting preoperative risk stratification and multidisciplinary surgical planning—for example, consistent with TVU-based ultrasound scoring approaches for identifying high-risk EC patients (26)—rather than as a direct decision tool for or against lymphadenectomy.

The clinical implications of our findings are potentially meaningful for preoperative risk stratification and surgical planning discussions. Patients in the highest ET tertile (>30 mm) faced a 49.0% LNM rate, whereas those with ET < 20 mm had a rate of only 2.8%. The proposed nomogram may serve as a preoperative decision-support tool to facilitate individualised risk communication and multidisciplinary surgical planning. Patients with high predicted LNM probability—for example, those with ET above the 28.5 mm threshold combined with elevated CA-125, NLR, or fibrinogen and a low PNI—may be prioritised for sentinel lymph node mapping or systematic lymphadenectomy as part of their surgical staging plan. Conversely, patients with low predicted risk, including those with ET < 20 mm, normal nutritional, inflammatory, and metabolic indices, and grade 1 endometrioid EC, may represent a group in whom the benefit of extended nodal staging is limited. It is emphasised, however, that the nomogram should complement rather than replace clinical judgement and intraoperative findings, and that prospective external validation is required before routine clinical implementation.

Several limitations warrant acknowledgment. As a single-centre retrospective study conducted at a tertiary gynaecological oncology referral centre, the LNM rate of 17.2% is higher than that reported in some population-based series, likely reflecting an enriched high-risk surgical case mix. This may limit the generalisability of the model to institutions with a lower background LNM prevalence, and recalibration would be required in such settings. Selection bias inherent to the retrospective design cannot be fully excluded. The sample size (*n* = 203), while adequate for the number of predictors evaluated, limits the precision of subgroup estimates. With 35 LNM-positive events and eight predictors, the events-per-variable ratio of approximately 4.4 falls below the conventionally recommended threshold of 10, and the risk of overfitting cannot be dismissed despite bootstrap internal validation. The optimism-corrected C-index, LASSO sensitivity analysis, and calibration plot are provided to enhance transparency of model performance, but prospective external validation in independent, geographically diverse cohorts remains an essential prerequisite before clinical implementation. We did not include MRI or sentinel lymph node biopsy data, both of which provide complementary information. The TVU assessments were performed by multiple sonographers, and inter-observer variability in ET measurement, though minimized by standardized protocols, was not formally quantified. Molecular classification data—including POLE mutational status, mismatch repair (MMR) proficiency, and p53 immunohistochemistry—were not systematically available in this retrospective cohort. This represents a substantive limitation, given that the 2021 ESGO/ESTRO/ESP guidelines and the updated 2023 FIGO staging system assign molecular subtype a central role in risk stratification: POLE-ultramutated tumours carry a favourable prognosis even in the presence of adverse morphological features, while p53-abnormal tumours are associated with a high risk of advanced disease and lymph node involvement. The proposed nomogram must therefore be understood as a complementary preoperative decision-support tool and must not substitute for molecular classification where it is clinically available. Future prospective studies should prospectively incorporate molecular subtyping alongside TVU morphological and nutritional-inflammatory-metabolic parameters. Importantly, recent evidence confirms that microsatellite instability (MSI) and high tumour mutational burden (TMB) detected by next-generation sequencing show high concordance with loss of mismatch repair proteins by immunohistochemistry (27), supporting the feasibility of integrating NGS-based molecular profiling into the preoperative assessment workflow alongside the imaging and nutritional indicators incorporated in our nomogram. Longitudinal follow-up data on recurrence and survival were not included in the present analysis. Finally, causal inference between nutritional, inflammatory, and metabolic status and LNM risk cannot be established from a retrospective design. Future prospective studies incorporating serial nutritional biomarker assessment alongside multi-omics profiling would strengthen mechanistic interpretation. In particular, the integration of circulating cell-free DNA methylation profiling—recently validated across multiple cancer types as a promising non-invasive biomarker platform (28)—with preoperative TVU and nutritional-inflammatory-metabolic indicators may open new avenues for minimally invasive LNM risk stratification in EC.

## 5 Conclusion

Preoperative TVU-measured endometrial thickness is significantly and broadly correlated with nutritional, inflammatory, and metabolic indicators—including CA-125, HE4, NLR, PNI, fibrinogen, albumin, and the TyG index—in patients with endometrial cancer. ET, in conjunction with deep myometrial invasion, CA-125, NLR, PNI, fibrinogen, tumor grade, and histological type, independently predicts lymph node metastasis preoperatively. A nomogram integrating these parameters achieved promising, good internally validated discriminative performance (AUC = 0.941) and may serve as a practical preoperative tool for individualized lymph node metastasis risk stratification, guiding surgical planning and personalized management of endometrial cancer. The proposed nomogram is intended as a complementary tool within a comprehensive preoperative assessment framework that includes molecular subtyping where available, and prospective external validation is required before clinical implementation.

## Data Availability

The datasets analyzed during the current study are available from the corresponding author on reasonable request, subject to applicable ethical and privacy restrictions.
